# Putting post-decision wagering to the test: a measure of self-perceived knowledge in basic sciences?

**DOI:** 10.1007/s40037-019-0495-4

**Published:** 2019-02-05

**Authors:** Marjolein Versteeg, Paul Steendijk

**Affiliations:** 1 Center for Innovation in Medical Education, Leiden University Medical Center, Leiden, The Netherlands; 2 Department of Cardiology, Leiden University Medical Center, Leiden, The Netherlands

**Keywords:** Medical education, Instructional methods, Quantitative Research Methods, Metacognition

## Abstract

**Introduction:**

Students learn more effectively when they know what they do not know. Gaining insight into students’ metacognitive awareness is needed as misalignment between actual and self-perceived knowledge impedes their learning process. The optimal method of measuring self-perceived knowledge is still under debate. In this study, we evaluate the use of psychology-derived post-decision wagering for mapping students self-perceived knowledge.

**Methods:**

Students (*n* = 71) performed a pre-test on medical physiology, followed by a teacher-moderated discussion and a post-test with isomorph questions. Half of the students rated their self-perceived knowledge on each question using post-decision wagering, i. e. betting 1–5 points on the correctness of their answer, whereas the other half used a 5-point Likert scale to rate their confidence.

**Results:**

Self-perceived knowledge scores were higher for post-decision wagering (pre: 3.75 ± 0.14, post: 4.60 ± 0.07) compared with Likert scales (pre: 3.13 ± 0.08, post: 3.92 ± 0.08) despite similar actual knowledge scores. Furthermore, Likert ratings showed a near-normal distribution, whereas wagers were placed preferentially using the outer ends of the scale. Correlations between mean actual and self-perceived knowledge scores were low in both groups. On average, 8.5% of responses were classified as misconceptions, defined as highly confident incorrect answers.

**Discussion:**

Despite the presumed reliability of post-decision wagering, our findings suggest that we should adhere to the use of Likert scales as a balanced measure for self-perceived knowledge in medical education. Moreover, the prevalence of misconceptions did not alter after instruction, indicating a need for instructional designs that enhance students’ conceptual understanding in basic sciences.

## What this paper adds

The importance of ‘knowing what you do not know’ is well-acknowledged in medical education. However, the optimal method of measuring self-perceived knowledge is still under debate. In this study, a psychology-derived tool to measure self-perceived knowledge called post-decision wagering was evaluated and considered less effective for educational settings than traditional Likert scales. We recommend including Likert scales in multiple-choice formats to establish confidence-weighted practice tests. Such tests may inform both students and educators on the student’s level of understanding, making it a more powerful tool for improving teaching and learning.

## Introduction

Learning basic sciences in medicine is recognized as an important, but challenging undertaking. It requires students to achieve understanding of complex learning material by analyzing, conceptualizing, and integrating knowledge. Strikingly, students are often unaware of what they do and do not know, which can be detrimental to learning [1, 2]. We cannot expect students to perform as effective learners if they are unaware of their own knowledge deficiencies.

Being aware of knowledge deficiencies is considered part of one’s metacognition. Metacognition, put simply, refers to thinking about one’s own thinking [3]. The ability to evaluate one’s knowledge (i. e. self-perceived knowledge) after performing a task or test (i. e. actual knowledge) is a subcomponent of metacognition, which we refer to as *metacognitive evaluation*: knowing how well you did after finishing a task [3–5]. Metacognitive evaluation is considered a critical component for learning as it informs both students and educators on students’ potential knowledge deficiencies [6, 7]. Moreover, some researchers have showed that including metacognitive evaluation through confidence marking during multiple-choice practice tests increases the predictive value of testing [8–10].

However, studies assessing metacognitive evaluation in medical education mainly focus on clinical knowledge [11–18], while the domain of basic sciences remains largely unexplored. Particularly within this latter domain, misunderstandings of physiological scientific concepts should be detected and corrected, since these concepts form a foundation for accurate clinical reasoning [19, 20]. Regarding basic science knowledge, Dawson et al. illustrate that according to faculty teachers physiological topics are experienced by medical students as being among the most difficult [21]. This finding is supported by research that has shown a high prevalence of misunderstandings among students in medical physiology education [22–25]. According to conceptual change theory, these so-called *misconceptions* are different from a mere lack of knowledge; misconceptions are robust to change and are therefore difficult to alleviate by traditional teaching methods [26]. Misconceptions are generally indicated by incorrect answers that are given by students with high confidence, indicating that students are unaware of their incorrect knowledge [27]. The role of awareness in establishing conceptual understanding illustrates that conceptual change theory and metacognitive theory are strongly intertwined in practice. Recognizing and evaluating existing conceptions, and deciding whether to reconstruct or review one’s understanding are all metacognitive processes [28]. Conceptual change theory emphasizes the key role of awareness in accomplishing the shift from a misconception to the scientifically correct conception [26, 29–31]. Awareness can be created by explicitly assessing students’ metacognitive evaluation in the classroom, as it forces both learners and educators to think about their understanding of the subject matter.

Research on science learning in higher education nicely illustrates how metacognitive evaluation can be measured in classroom settings using a multi-tier approach [32–34]. Multi-tier assessments consist of multiple-choice exercises with a complementary Likert scale question: ‘How confident are you that your answer to this question is correct?’. This is an example of measuring metacognitive evaluation; assessing students’ actual knowledge through test scores and their self-perceived knowledge through Likert scales. The use of Likert scales as a self-report tool for self-perceived knowledge is very common in educational research, with variations in scales ranging from 3 to an increasing number of integers [11–15, 32–35]. However, there is some controversy on the objectiveness of Likert scales for measuring self-perceived knowledge. As noted in a review by Koch and Preuschoff: ‘Studying consciousness is difficult because asking subjects to report their awareness of a stimulus perturbs that awareness’ [36]. In psychology research, an alternative method to more implicitly measure awareness of visual stimuli was introduced, called post-decision wagering (PDW) [37]. This method is suggested to determine awareness more objectively by indirectly assessing individuals’ self-perceived knowledge by betting points (or money) on their decisions. If an individual chooses the correct answer points are gained, whereas with a bet on an incorrect answer points are lost. Additionally, researchers suggest that PDW enhances individuals’ motivation to reveal their confidence in their answer compared with self-reports on numerical confidence scales [38, 39]. Contrastingly, various studies show a substantial influence of loss aversion in PDW resulting in a decreased accuracy towards measuring self-perceived knowledge. Therefore, participants are less prone to indicate a high confidence level through wagering [40, 41]. Moreover, wagers are also suggested to depend on wager size indicating that PDW is still a subjective tool to measure awareness [42].

The use of PDW as a tool to measure self-perceived knowledge has not yet been reported in medical education. In this study, our primary objective was to evaluate psychology-derived PDW as a measure of self-perceived knowledge in educational contexts. We compared PDW with Likert scales and hypothesized that confidence wagers rather than ratings would be more aligned with students’ actual knowledge due to their supposed more objective nature. To further determine the practical usefulness of the instruments in quantitating self-perceived knowledge, we examined the distribution of responses along the scales to compare effective resolution and discriminative power. As a secondary objective, we investigated the prevalence of misconceptions among students to gain insight into their conceptual understanding of the subject matter. In the present study, we focused on basic sciences particularly given the high prevalence of misconceptions [21–25, 43]. Misconceptions may not only affect students’ conceptual understanding, but also have significant impact on their clinical reasoning skills [23, 44, 45], and are therefore considered an important subject of investigation in medical education.

## Methods

### Participants

A cohort of first-year bachelor Biomedical Sciences students (*n* *=* 71) from Leiden University Medical Center participated in this study. In the Netherlands, similar physiology courses are part of both the biomedical and medical bachelor curriculum.

### Setting

For this study, the online platform *Socrative* was used to develop multiple-choice knowledge tests. The knowledge tests were implemented in a compulsory 2‑hour supervised seminar on cardiovascular physiology that was taught in five small working groups (13-15 students/group) by the same teacher. Allocation of the students to the working groups was arbitrary except for the aim to have a similar female/male ratio in all groups. The topics were introduced in a preceding plenary lecture, and students were provided with a handout during the seminar which contained some basic figures and diagrams. Although the knowledge tests were a mandatory part of the seminar, students could voluntarily decide if their anonymous answers could be used for educational research purposes by giving informed consent. No incentives were offered for participation and test results did not affect the course grade. Students could withdraw their permission at any time. This experiment was part of a larger study also investigating the influence of peer discussion on confidence of which data are not reported in this paper. This study was approved by the Educational Research Review Board (ERRB) of Leiden University Medical Center: ERRB reference number: OEC/ERRB/20180612/3.

### Procedure

The knowledge tests were taken individually under exam conditions, at the beginning (pre) and at the end (post) of the seminar. Both tests consisted of 10 multiple-choice questions and examined the same knowledge base. The post-test used slightly different phrasing of the questions which can be considered isomorphic or so-called near-transfer questions. All students were instructed to answer each question individually and to provide a confidence rating (Likert scale: 3 out of 5 groups, *n* = 42) or wager (PDW: 2 out of 5 groups, *n* = 29) immediately after each question. To compute the actual knowledge, students received one point for every correct answer of the multiple-choice tests yielding a maximum score of 10 points per test. During the body of the actual seminar the questions from the pre-test and related topics were explained and discussed.

Questions were framed according to the revised version of Bloom’s taxonomy of cognitive domain and all categorized as comprehension-type questions. To ensure validity of the knowledge test, the physiology questions were designed by an expert physiologist (PS) and derived from a database of previously used exam questions. PS is an educational professor at Leiden University Medical Center and actively involved in shaping the biomedical and medical curriculum regarding medical physiology education, and familiar with the learning goals of the human biology course. Consequently, the questions were designed in such a way that they would meet the overall course objectives regarding physiological knowledge.

### Instruments

For confidence rating in the Likert scale group (LS group), students used a 5-point Likert scale rating instrument: (1) Completely unsure (just guessing), (2) Rather unsure, (3) Neutral (50/50), (4) Rather sure, (5) Very sure (almost 100%). Students received additional instruction on the neutral item (3) as the lecturer indicated that this rating should be given when a student doubted between two remaining answer choices considered equally likely. In the PDW group, students were instructed to place their bets on the correct answer. In order to compare results with the LS group, wagers ranged from 1 to 5 points. Students could bet 1–5 points per question, which were gained if the answer was correct and lost if the answer was incorrect. Students received their total wagering scores after the seminar.

### Data analyses

**Fig. 1:**
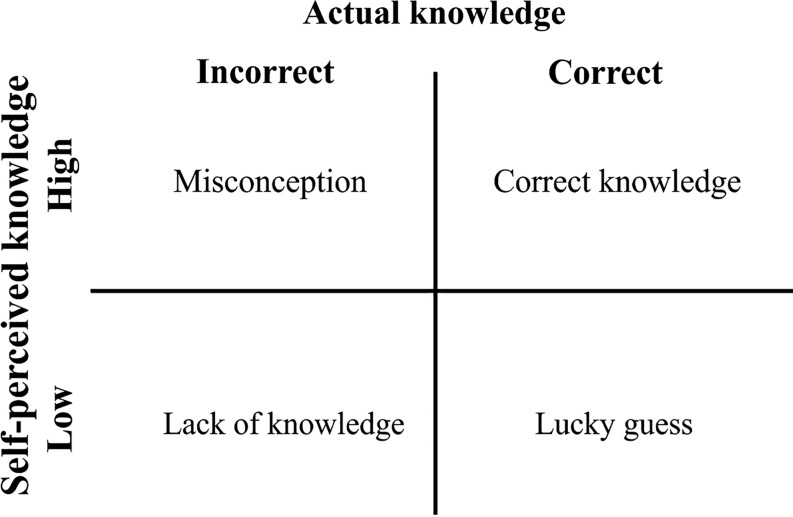
A decision matrix based on students’ actual and self-perceived knowledge. Adapted from [27]

Descriptive statistics are provided as means and standard errors of the mean, unless otherwise mentioned. Actual and self-perceived knowledge scores in PDW and LS groups were compared using independent samples t‑tests. Comparison of score gains on the pre-post-test between groups was performed with a repeated measures ANOVA. To test and compare alignment between actual knowledge and self-perceived knowledge scores, correlation analysis was performed using Spearman’s rank order correlation coefficient. Scores were averaged per student. A Fisher’s r‑to-z transformation was performed to test for potential significant differences between correlations. The discriminative power of both methods was tested by performing Chi-square tests of independence. Hasan’s decision matrix was used as a template to analyze the prevalence of misconceptions (Fig. 1; [27]). To create dichotomous outcomes, a threshold value of 3 was adopted and used as a cut-off point. Students were instructed to rate an answer with 3 when they were in doubt between two answer choices considered equally likely, meaning that they were still unsure about knowing the correct answer, i. e. having low confidence. Correct answers self-rated with a high confidence (>3) were classified as ‘correct knowledge’ and when rated with low confidence (≤3) as ‘lucky guess’. Incorrect answers with a low confidence rating were considered ‘lack of knowledge’ and when paired with high confidence, the response indicated the presence of a misconception. IBM SPSS Statistics Version 23.0 (IBM Corp., Armonk, New York, USA) and GraphPad Prism Version 7.02 (GraphPad Software, La Jolla, California, USA) were used for all data analyses and visualizations.

## Results

The average confidence scores reported in the PDW group on the pre-test and post-test were significantly higher compared with the scores in the LS group (both *p* < 0.001) (Tab. 1). The gain in confidence scores in the PDW group (0.86 ± 0.12) was significantly higher than in the LS group (0.79 ± 0.12) (*p* *<* 0.001, η^2^ = 0.349). The average test scores did not differ significantly between PDW and Likert scale on the pre-test (*p* = 0.338) and post-test (*p* = 0.065). In the PDW group, students showed an average increase of 1.55 ± 0.30 points in their pre- to post-test scores, which was not significantly different from the gain of 1.32 ± 0.28 points in the LS group (*p* *=* 0.094).

**Table 1:** Mean scores for students’ actual and self-perceived knowledge

Instrument	Actual knowledge (Score, max 10)	Self-perceived knowledge (Score, max 5)	No. of students
*Pre-test*			
Post-decision wagering	6.78 ± 0.27	3.75 ± 0.14	29
Likert scale	6.38 ± 0.27	3.13 ± 0.08	42
*Post-test*			
Post-decision wagering	8.31 ± 0.23	4.60 ± 0.07	29
Likert scale	7.68 ± 0.27	3.92 ± 0.08	41

### Alignment

**Fig. 2:**
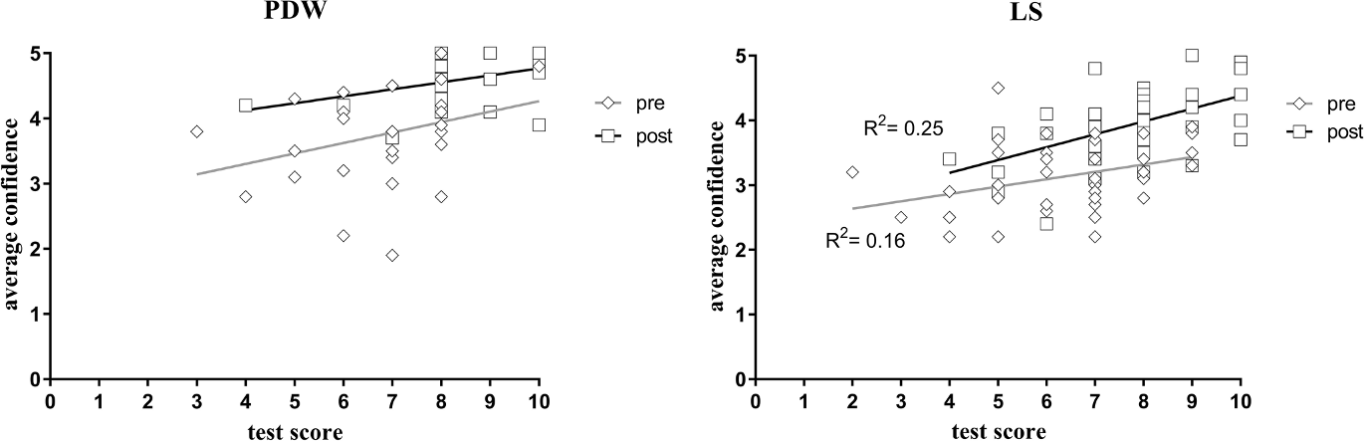
Relationship between actual and self-perceived knowledge of students

There was a significant correlation between actual and self-perceived knowledge scores in the LS group on the pre-test (R^2^ = 0.16, *p* < 0.01) and post-test (R^2^ = 0.25, *p* *=* 0.001) (Fig. 2). In the PDW group correlations between actual knowledge and self-perceived knowledge are absent on both tests (pre: R^2^ = 0.12, *p* = 0.063, post: R^2^ = 0.10, *p* = 0.102). To test for potential differences between the correlations in the PDW and LS group for the pre-test and post-test respectively, correlations were transformed into z‑scores using Fisher’s r‑to-z transformation. The differences between correlations on the pre-tests (z = −0.23, *p* = 0.817) and post-tests (z = −0.90, *p* *=* 0.370) were not significant.

### Distribution

**Fig. 3:**
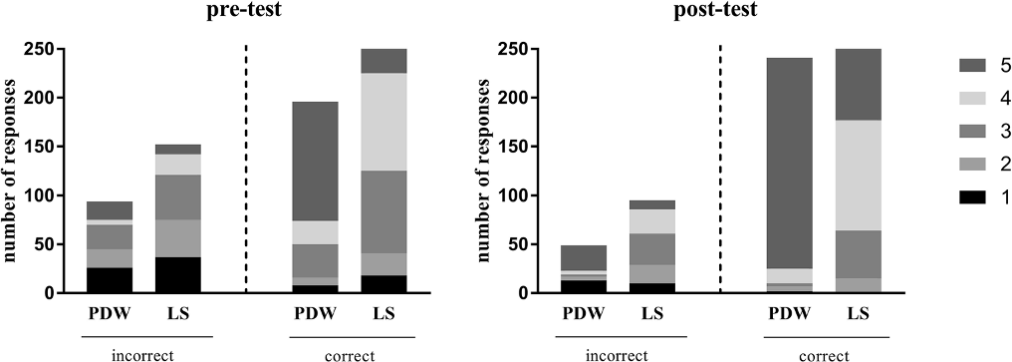
Distribution of self-perceived knowledge scores

The distribution of confidence scores (1–5) for the Likert scale and PDW instruments is shown in Fig. 3. Comparing the distribution of 1–5 responses on the pre-test yielded a significant difference in distribution in self-perceived knowledge scores between PDW and Likert scale on questions with an incorrect response (*p* < 0.007, φ = 0.240). Of all questions answered incorrectly in the PDW group, 25.5% of responses were rated with level 5 confidence, compared with only 6% in the LS group. An even more pronounced difference between the Likert scale and PDW was found for correctly answered questions, yielding 43.8% level 5 confidence scores in the PDW group versus 16.3% in the LS group (*p* < 0.001, φ = 0.483). Discrepancies in the self-perceived knowledge distribution between Likert scales and PDW were also obtained for the post-test. The largest effect size was obtained for incorrect answers on the post-test (*p* < 0.001, φ = 0.593) with 53.1% level 5 responses in the PDW group and 9.7% in the LS group. For correct answers, 89.6% was associated with confidence level 5 in PDW versus 43.6% in the Likert scale (*p* < 0.001, φ = 0.483).

### Misconceptions

**Fig. 4:**
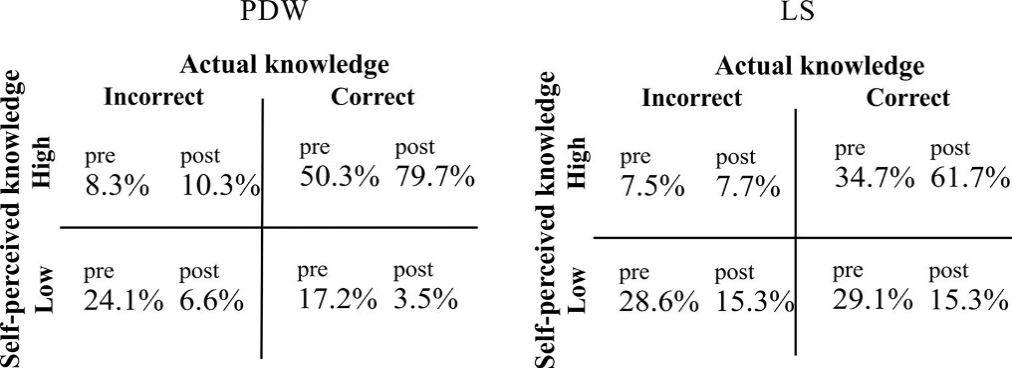
Prevalence of misconceptions

There was an increase in ‘correct knowledge’ in both groups after the plenary instruction and discussion (PDW: 50.3 to 79.7%; LS: 34.7 to 61.7%) (Fig. 4). The total number of ‘lucky guesses’ was reduced (PDW: 17.2 to 3.5%; LS: 29.1 to 15.3%) as was the amount of ‘lack of knowledge’ (PDW: 24.1 to 6.6%; LS: 28.6 to 15.3%). The prevalence of misconceptions was similarly present before and after instruction (PDW: 8.3 to 10.3%; LS: 7.5 to 7.7%).

## Discussion

The aim of this study was to evaluate psychology-derived post-decision wagering (PDW) as a measure of self-perceived knowledge by comparing it with the commonly used Likert scales. Despite similar levels of actual knowledge in both groups, students who used wagers indicated more confidence in their answers compared with students who used ratings. PDW confidence scores were also less evenly distributed compared with Likert scales and did not show a normal distribution, resulting in a less proportional use of the instrument. Contrary to our a priori hypothesis, PDW thus represents a less balanced measure of self-perceived knowledge than the traditional Likert scale.

Misalignment between actual and self-perceived knowledge was present in both groups, indicating that students generally do not know what they know or do not know. Some students using PDW mentioned that they would easily go ‘all in’ and bet all their points even when they were not completely sure, and despite the rule that points were lost in case of an incorrect answer. Contrary to these findings, psychological research and behaviour economics report that individuals who wager generally show lower confidence compared with verbal confidence reports due to so-called risk aversion [41, 42]. The apparent absence of risk aversion in our students might be due to the use of imaginary ‘points’ as incentives. Interestingly, however, studies in laboratory settings have reported similar responses when using real versus imaginary incentives [37]. We suggest that the educational environment in which students are socially engaged might influence their response to risk aversion. The effects of classroom versus laboratory settings on the use of self-perceived knowledge measures await further investigation.

Our findings are in line with previous studies reporting discrepancies between students’ actual and self-perceived knowledge consistently across disciplines [1, 6, 15, 24, 46–49]. Kruger and Dunning have suggested that incompetence deprives an individual of the ability to recognize its shortcomings [2]. Based on this reasoning, our reported underestimation of performance is an unexpected finding. An explanation for this discrepancy may be that metacognitive evaluation of the specific materials in this study did not resemble the more global self-assessments mentioned in Kruger and Dunning’s studies. Such global judgements are, for example, self-competence (‘I feel I am able to …’) and self-efficacy (‘how confident are you that you can …’). These are prospective judgements and can therefore be considered part of one’s metacognitive knowledge, whereas metacognitive evaluation (as measured in the present study) is an on-line judgement which is made after one has performed a specific task [4, 5]. Students might be more cautious with estimating their actual scores after they have performed a task compared with a situation in which a global prospective judgement is requested. Furthermore, gender differences may have influenced our outcomes. Research has indicated that women tend to underestimate their academic capacities, as illustrated by the study by Ravesloot on progress testing in medical education [50]. Most of the students (70%) in this experiment were female, which might partly explain the relatively large percentage of correct low-confidence responses.

Overall, teacher instruction during the seminar reduced the percentage of lack of knowledge and lucky guesses and increased the number of correct responses, whereas the number of misconceptions was not greatly altered. Based on conceptual change theory and schema theory, we assume that the seminar discussion may not have benefitted the students with misconceptions, as the instructional design to achieve conceptual change should include more explicit activation of students’ prior knowledge. A learner’s understanding is facilitated by adding new information to an existing mental model or schema in the brain, which comprises relevant prior knowledge a learner has already obtained [51–53]. Cognitive neuroscientists have shown that new information can be added to schemas (i. e. stable neural network) faster when this information fits the prior knowledge [54, 55]. Thus, when a learner’s schema encompasses a misconception, it may be difficult for the instructor to teach the scientific conception using traditional teaching methods. By activating students’ prior knowledge, their current understanding of concepts and potential misconceptions become detectable for instructors [56, 57]. Future studies may focus on the use of conceptual change instructions, including activation of prior knowledge, to alleviate the number of misconceptions and enhance students’ conceptual understanding.

Our study has several limitations. We focused on the comparison of different measurement instruments for self-perceived knowledge. The relatively low difficulty level of questions might have limited the prevalence of misconceptions as these were more comprehensive than conceptual-oriented questions. Additionally, the post-test questions were not identical but isomorph to the pre-test questions which may have resulted in unanticipated changes in item difficulty because of near-transfer conversions of the original items. Furthermore, students had limited time to process the information as the total length of a seminar was approximately 120 min. An additional retention test might have provided more insight into the stableness of students’ conceptual understanding.

## Conclusion

This study was conducted to evaluate post-decision wagering as an instrument for measuring students’ self-perceived knowledge in educational contexts. Our findings add to the growing number of studies that demonstrate the subjective properties of PDW and thus do not support previous literature on the objectivity of PDW as a measure of confidence. In all, this study supports the use of Likert scales over post-decision wagering as an instrument for measuring self-perceived knowledge in educational settings.
